# Enhanced sensitivity of surface plasmon resonance phase-interrogation biosensor by using oblique deposited silver nanorods

**DOI:** 10.1186/1556-276X-9-476

**Published:** 2014-09-09

**Authors:** Hung-Yi Chung, Chih-Chia Chen, Pin Chieh Wu, Ming Lun Tseng, Wen-Chi Lin, Chih-Wei Chen, Hai-Pang Chiang

**Affiliations:** 1 Research Center for Applied Sciences, Academia Sinica, Taipei 115, Taiwan; 2 Institute of Optoelectronic Sciences, National Taiwan Ocean University, Keelung 202, Taiwan; 3 Graduate Institute of Applied Physics, National Taiwan University, Taipei 106, Taiwan; 4 Institute of Physics, Academia Sinica, Taipei 115, Taiwan

**Keywords:** Surface plasmons, Biosensor, Nanorods, Oblique angle deposition, Phase interrogation

## Abstract

Sensitivity of surface plasmon resonance phase-interrogation biosensor is demonstrated to be enhanced by oblique deposited silver nanorods. Silver nanorods are thermally deposited on silver nanothin film by oblique angle deposition (OAD). The length of the nanorods can be tuned by controlling the deposition parameters of thermal deposition. By measuring the phase difference between the *p* and *s* waves of surface plasmon resonance heterodyne interferometer with different wavelength of incident light, we have demonstrated that maximum sensitivity of glucose detection down to 7.1 × 10^-8^ refractive index units could be achieved with optimal deposition parameters of silver nanorods.

## Background

Optical sensors, the devices which can transform the information of light-matter interactions into the analytic electronic signal, become more and more important nowadays for the fields such as security for environment and food [1,2], energy [3], remote sensing, and medical diagnostics [4] because of their ultrafast responsibility and remote sensing ability. Among the numerous optical sensors, plasmonic nanosensors have great promise due to their spectral tunability and much better adaptability to modern nanobiotechnologies. Surface plasmon resonance (SPR) as a kind of electromagnetic resonance of the conduction electrons on metal surface is very sensitive to the environmental refractive index variation, which can be considered as a useful sensing parameter [5]. For the plasmonic sensing applications, using the so-called Kretschmann geometry [6,7] to excite the SPR at the metal/target analyte interface is very fascinating because of its low-cost, compact, and flexible experimental setup. Being launched under the Kretschmann geometry, the spectral reflection dip of SPR mode can be monitored to follow any changes that take place in the proximity [8]. Generally speaking, there are several choices of parameters that can be used to accomplish the SPR sensing process based on Kretschmann geometry.

Since the first observation of surface plasmon resonance (SPR), various optical methods have been explored to excite SPR at a metal-dielectric interface [9]. Such an excitation can be utilized to achieve sensing of various interfacial phenomena with extreme sensitivity. These include, for example, chemical and biological sensing [10-18], film-thickness sensing [19], temperature sensing [20], and angular measurement [21]. Among various plasmonic sensing techniques, it has been quite well-known that the ‘phase interrogation’ technique is by far the most sensitive one in many applications [11,13-17,19,20]. Recently, we have found that the wavelength of incident light will affect the detection sensitivity in the SPR monitoring of temperature [10]. We also observed that high-resolution angular measurement and biological sensing can be achieved by SPR phase interrogation at optimized incident wavelength [17,20]. However, the value of the optimal wavelength will change when the film thickness varies. It is thus possible to reach optimal sensitivity by tuning the thickness of metallic film at fixed wavelength of incident light. Furthermore, modification of the surface roughness of the metallic film will also change the sensitivity of SPR sensor [9,21,22].

**Figure 1:**
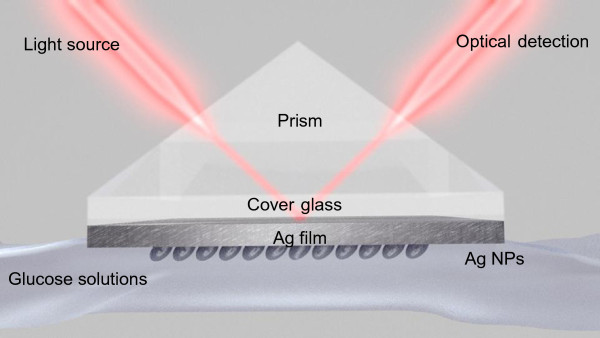
Schematic picture of the SPR biosensor.

In this paper, we demonstrate that the sensitivity of the Kretschmann-based plasmonic sensor shown in Figure 1 can be further improved through the use of the oblique angle deposition (OAD) technique [23,24] to fabricate the oblique Ag nanorods on the precoated Ag thin film. The OAD technique as a kind of high-throughput nanofabrication technology is very useful in various applications of nanophotonic components such as achromatic waveplates [25], metamaterials with negative refractive index [26], and antireflection coating [27]. The range of tilt angle between the nanorod and the Ag film can be controlled by varying the vapor incident angle [28]. J. Fu et al. also discussed the reflectance of nanorod-based SPR sensor by using effective medium theory. Comparison between theoretical calculations and experimental results are fully discussed [28]. In this article, we have discussed the optimization conditions of the sensitivity of SPR phase-interrogation biosensor enhanced by nanorods fabricated by OAD method operated at different incident wavelengths.

## Methods

**Figure 2:**
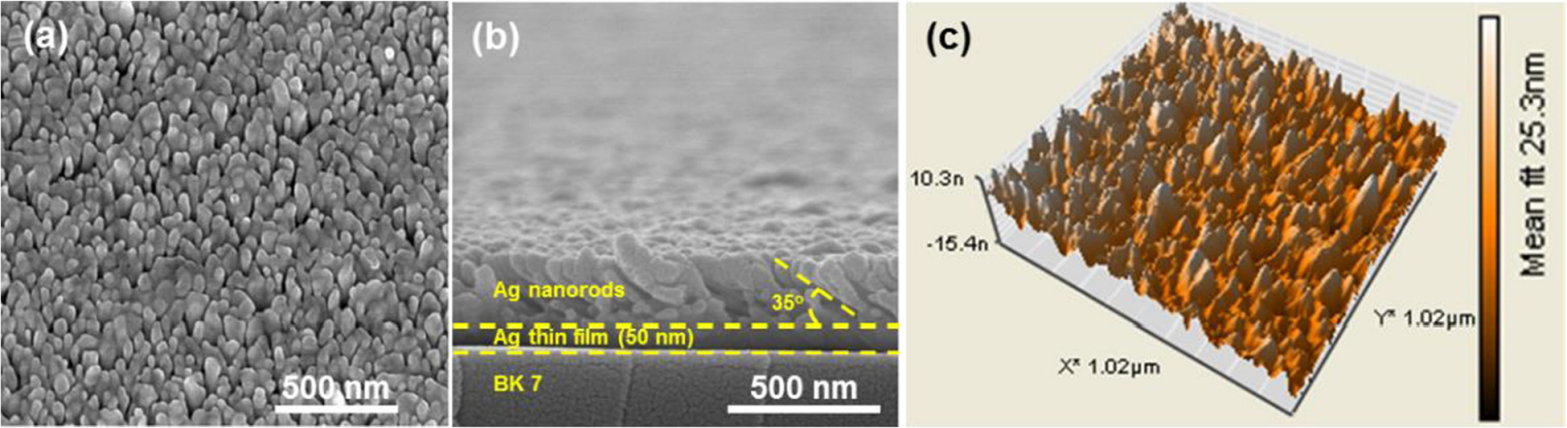
**Images of the Ag nanorods. (a)** Top-view SEM image of the Ag nanorods. **(b)** Cross-sectional-view of the Ag nanorods. **(c)** Three-dimensional morphologic AFM image of the Ag nanorods.

For fabrication of the Ag nanostructures, at first the 50-nm-thick Ag thin film is deposited on the cleaned transparent BK7 glass substrate (Matsunami cover glass, 22 × 22 mm^2^, 0.15-mm thickness, Matsunami, Kishiwada, Osaka, Japan) by thermal deposition method under Ar pressure of 5 × 10^-6^ Torr (deposition rate approximately 0.5 Å/s). Subsequently, the vapor incident angle between the substrate and horizontal line is set as 85°, which means the tilting angle between the substrate and Ag source is 5° for generating the tilted Ag nanorods on the Ag thin film. Figure 2a is the scanning electron microscope (SEM) image of the fabricated Ag nanostructures. In the SEM image, irregular Ag nanoparticles are observed. Some Ag nanoparticles are aggregated into the cluster, which may be associated to the self-aggregation behavior of the cooling down Ag materials in deposition. The sizes of the Ag nanostructure range from around 50 to 200 nm. As shown in Figure 2b, which is the cross-sectional SEM image of the fabricated nanostructure, the substrate, Ag thin film with thickness of 50 nm, and the tilted Ag nanorods can be clearly distinguished. The tilt angle between the nanorod and the Ag film is around 35° ± 5°, which is larger than the tilting angle between the substrate and Ag source. This result is similar to the results reported by Y. J. Jen et al*.*[26]. The average length and diameter of the nanorods are around 300 and 50 nm, respectively. Similar observation result of surface morphology can be obtained under the atomic force microscope measurement (Nanosurf Mobile S, AFM, Nanosurf, Liestal, Switzerland) in contact mode (Figure 2c). In AFM image, the random distribution of the Ag nanorods can be observed, and the variation of the height is around 25.3 nm.

## Results and discussion

The experimental setup is identical to that used in [13], except the Ag nanoparticles are replaced by the Ag nanorods shown in Figure 2.

**Figure 3:**
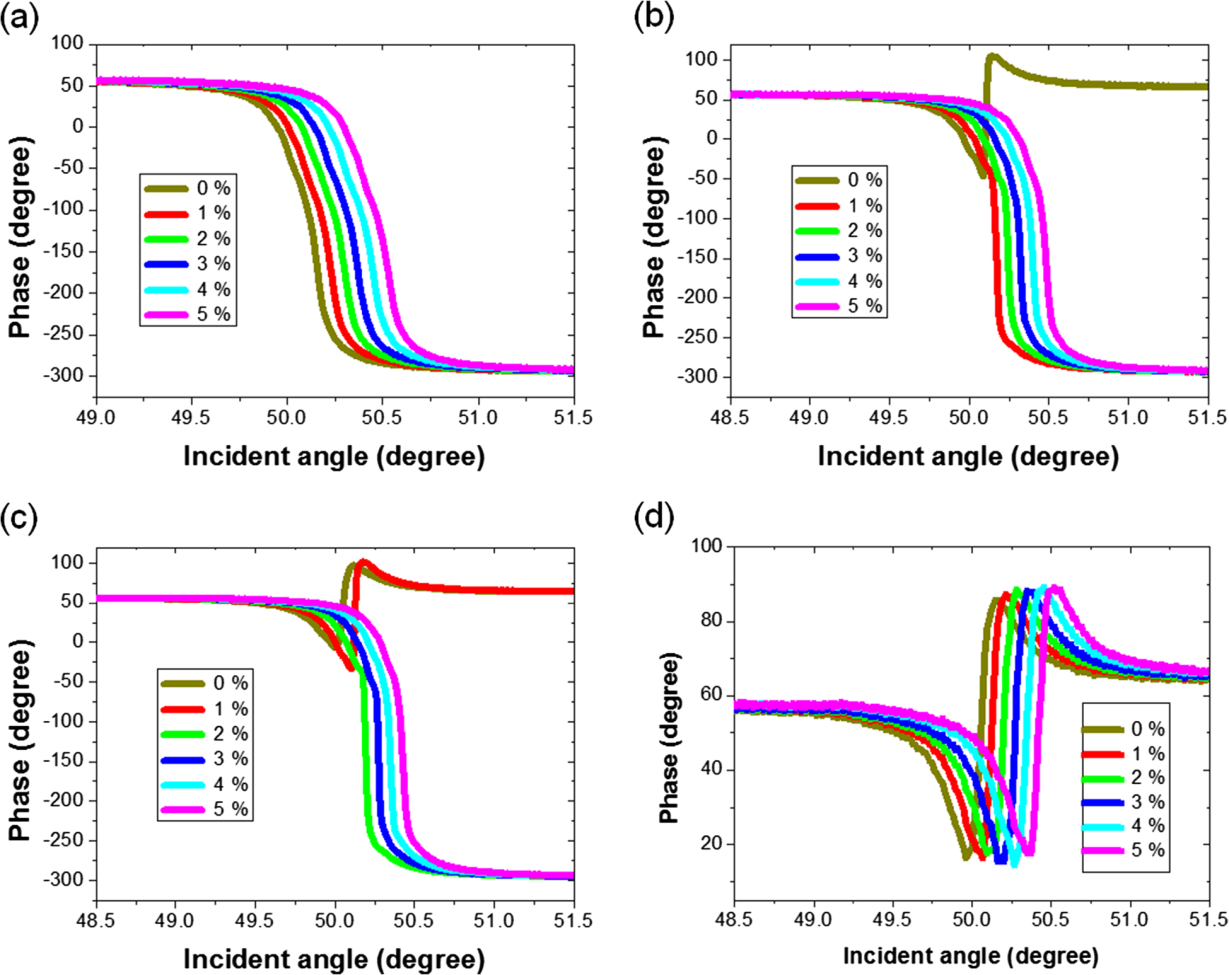
**Experimental results of the relative phase difference between the *p *and the *s *waves.** With different incident angles for various glucose solutions. The incident wavelength is fixed at 1,150 nm. The SPR sensing chips employed are oblique silver nanorod thin films of the thickness **(a)** 6, **(b)** 8, **(c)** 10, and **(d)** 12.5 nm, respectively.

Figure 3 shows the measured results of the phase difference between the *p* and *s* waves of the wavelength 1,150 nm. Four different thicknesses, 6, 8, 10, and 12.5 nm, of the nanorod thin films are considered. As we can see, the resonance angle increases as the concentration of the glucose solution increases. This is a consequence of the SPR taking place, and satisfying the condition

(1) $$k_{∥}=k_{\text{sp}}\text{,}$$

where $k_{∥}=\left(\omega /c\right)\sqrt{\varepsilon_{p}}\sin\theta$ is the parallel component of the incident wave vector with the dielectric function of prism *ε*_*p*_ and the incident angle *θ*, and *k*_*sp*_ is the wave vector of the SP waves. Though *k*_*sp*_ is very complicated due to the surface roughness of the nanorod thin film, it can be roughly approximated by the form of the SP wave on a plane,

(2) $$k_{\text{sp}}\approx \frac{\omega }{c}\sqrt{\frac{\varepsilon_{\text{rod}}\varepsilon_{g}}{\varepsilon_{\text{rod}}+\varepsilon_{g}}}\text{,}$$

where *ε*_*rod*_ is the effective dielectric function of the nanorod thin film and *ε*_*g*_ is the dielectric function of the glucose solution. In our samples, the nanorod thin films are almost filled by silver, so *ε*_*rod*_ can be roughly estimated by the dielectric function of silver *ε*_*Ag*_ = -61.35 + 4.18*i* at the working wavelength *λ* = 1,150 nm [29]. On the other hand, since the concentration of the glucose solution is very low, the refractive index can be expressed by a linear relation [30]

(3) $$n_{g}=\sqrt{\varepsilon_{g}}\approx n_{w}+\alpha b\text{,}$$

where *n*_*w*_ = 4/3 is the refractive index of water, *α* is the concentration of the glucose solution, and *b* is some dimensionless fitting constant. Substituting Equations 2 and 3 into Equation 1, one can verify that the resonance angle increases as the concentration increases.

Another feature shown in Figure 3 is that there are two types of the phase change near the resonance angle, see Figure 3a,d. For the thinner nanorod film, the phase changes from higher to lower values while the reverse takes place when the nanorod film becomes thick. These behaviors are caused by the competition between the two damping rates: the internal damping and the radiation damping [10,31]. For the thinner nanorod films, the energy of the SP waves are mainly dissipated via radiation damping, in which the EM fields are coupling backward to the prism side and then radiate to space. When the film thickness increases, the internal damping dominates and the energy transfers to the thermal loss in silver. Moreover, Figure 2b,c shows that though the dominating damping is mainly determined by the film thickness, it also slightly depends on the concentration of the glucose solution.

**Figure 4:**
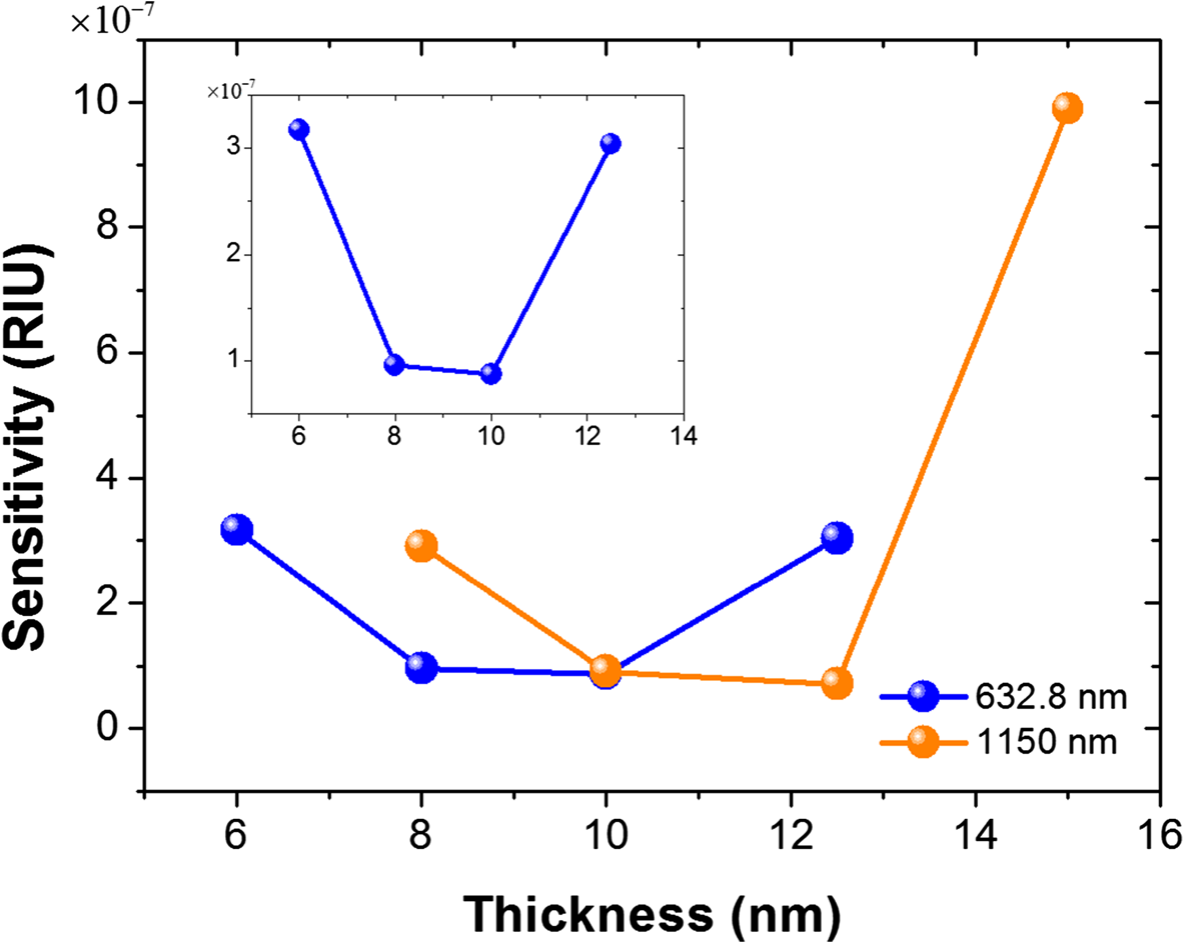
**Sensitivity as a function of the thickness at different incident wavelengths indicated in the figure.** Inset is the zoom in of the 632.8-nm case.

In order to seek the optimal thickness of the nanorod layer, we follow the definition of Nelson [12] to consider the quantity: *σ*_*n*_ = (*Δn*/*Δϕ*)*σ*_*ϕ*_, which indicates the smallest change of the refractive index that can be resolved by the measuring instrument, where *Δn*/*Δϕ* is the change of the refractive index per unit phase difference, and *σ*_*ϕ*_ is the finest resolution available of the measuring instrument, which is 0.01° from the lock-in amplifier used in our experiment. The refractive index of different glucose concentrations can be calculated by using the Fresnel equations [9]. Because this quantity indicates the smallest change of the refractive index that can be resolved by the measuring instrument, it can be treated as the definition of the sensitivity of the SPR sensor.Figure 4 shows the sensitivity of the nanorod thin film as functions of thickness. Except the original wavelength (1,150 nm) used in Figure 3, we also consider a short wavelength, 632.8 nm. As one can see, the thickness with the best sensitivity is around 10 nm in both wavelengths. The reason is that the enhancement of the sensitivity is due to the strong local field caused by the surface roughness, while the wavelength plays a minor role in this system.

The optimal thickness can be treated as a result of the balance between two factors: the coupling efficiency of the fields between the prism side and the solution side and the local field enhancement due to the surface roughness of the nanorod thin film. Nanorods thicker than the optimized thickness, the field coupled from the prism side of the Ag film is weaker, and thus reduce the strength of the field in the solution side and leads a lower sensitivity. On the other hand, if the thickness of the nanorod film is thinner than the optimized thickness, the surface becomes smooth and thus suppresses the local field enhancement. Therefore, the optimal thickness is a trade-off of the thickness that provides enough surface roughness to support the high concentrated local fields and also keeps the coupling effect strong enough.

Similar results can be observed from our previous study of the nanoparticle system [13]. In that system, the better sensitivity can be obtained when the sizes of the nanoparticles reduce. This is also due to the strong coupling efficiency from the prism side to the solution side. One can note that the performance of the nanoparticle system is overall better than the nanorod system. The reason is that because the size of both nanoparticles and nanorods in our study is much smaller than the wavelength, and the resonances of these nanoobjects are dominated by the dipole mode. It is well-known that the nanoparticle has a spherical symmetry, and hence is a better ‘container’ of the dipole mode.

## Conclusions

In this study, we have demonstrated the capability of the oblique deposited Ag nanorods as a SPR sensing chip. The sensitivity is dominated by the thickness of the nanorods, while the dependence of the incident wavelength is relative weak. Experimental results show that with the optimal thickness 10 nm of the nanorods, the sensitivity down to 7.1 × 10^-8^ can be achieved. Since the enhancement of the sensitivity is due to the strong local fields, the optimal thickness of the nanorod film comes from the balance between the coupling efficiency and the surface roughness.

## Competing interests

The authors declare that they have no competing interests.

## Authors’ contributions

HYC drafted the manuscript. CCC carried out the whole experiments. PCW and MLT participated in AFM and SEM measurements. WCL participated in the fabrication of nanorods. CWC participated in SPR phase-interrogation measurements. HPC designed the experiments, revised the manuscript, and approved the final version. All authors read and approved the final manuscript.
